# Promoting or Opposing Social Change: Political Orientations, Moral Convictions and Protest Intentions

**DOI:** 10.5964/ejop.v15i4.1693

**Published:** 2019-12-19

**Authors:** Stefano Passini

**Affiliations:** a Department of Education Studies, University of Bologna, Bologna, Italy; Georgetown University, USA

**Keywords:** protest intentions, SIMCA, political orientations, moral convictions, values

## Abstract

The issue of the motivations behind the decision as to whether or not to join protest actions has been investigated by many scholars. In particular, recent studies have considered violations of one’s own moral convictions and identification with the protest group as the main predictors of collective actions. The present research will focus on the three orientations to the political system identified by Kelman and Hamilton (1989), which consider distinct reasons behind the attachment to the political system and explain the motivations behind supporting or opposing the institutions. The aim is to examine whether these three orientations have an effect on collective action (through moral convictions, politicized identification, anger, and efficacy) considering social protests both against and in favor of the status quo. Specifically, the political orientations should explain why individuals hold a given attitude (positive or negative) towards a policy position, hold it with moral conviction, and decide to join a protest action. The results of three studies confirm the relevance of considering political orientations. Depending on the aim of the protest, each political orientation has a distinct effect on collective action.

In recent times, the growth of many protest and disobedience movements around the world within totalitarian regimes as well as democratic countries has led many scholars of social psychology (see van Zomeren, Postmes, & Spears, 2008) to inquire as to why some people decide to join these movements while others do not. Indeed, people often support movements that may prove to be costly (i.e., in terms of personal time, money, and risks) and from which they do not accrue rational benefits even in the event of a successful outcome (e.g., movements for the rights of Third World countries). Moreover, not all these protest movements are against the institutions and authorities in power and seek to promote social change. Many protest demonstrations defend the status quo and oppose social changes as well. For instance, due to the large wave of new immigration towards industrialized Western countries, some movements have expressed disapproval as concerns the hosting of migrants (Willis-Esqueda, Delgado, & Pedroza, 2017). This is the recent case of the anti-Islamic German organization Pegida which held rallies in 2016 in several European cities to protest against mass immigration and so-called “Islamization” of Europe. Similarly, in opposition to the broadening of civil rights to homosexual couples, some people have protested in defense of the so-called traditional family. For example, in 2016 thousands of protesters demonstrated in Paris against same-sex marriages and in support of those politicians who defend long-established family values.

Recent research has underlined the relevance of the psychology of collective action towards understanding the motivations behind the support to a protest (van Zomeren, Postmes, Spears, & Bettache, 2011). In particular, theories of social identity and social identification (see Simon & Klandermans, 2001; van Zomeren et al., 2008) consider identification with a social movement organization as the main predictor of collective action. However, although identification with the protest group does play a relevant role, considering what the vision of the society is which leads to the decision to join in a protest may be helpful for understanding social stability/social change dynamics. The recent extension of the Social Identity Model of Collective Action (SIMCA, see van Zomeren et al., 2011; van Zomeren, Postmes, & Spears, 2012) highlights how violations of moral convictions can motivate collective action by increasing identification with the protest group. Nevertheless, moral convictions are measured as a violation of a specific moral norm related to that collective action and the inclusion of this variable does not really explain why people hold such moral convictions in the first place, as also suggested by some research (e.g., Wisneski & Skitka, 2017).

The novelty of the present study is to consider those individual beliefs and motivations that lead people to view some policies as a violation of the relationship between the individual and the political system and to engage in collective actions. Specifically, the present research will focus on the three orientations to the political system (namely, rule, role, and value) identified by Kelman and Hamilton (1989) which consider distinct reasons behind the attachment to the political system and that explain the motivations behind supporting or opposing the institutions. The aim of the present research is to examine whether these three orientations have an effect on collective action (through moral convictions, politicized identification, anger, and efficacy) considering social protests both against and in favor of the *status quo*. The context of the research is Italy, a European country where the issue of minority rights (e.g., homosexuals, immigrants, and women) is constantly at the center of media, public and political debates. In the next paragraph, the SIMCA is presented. After that, the three political orientations are introduced.

## The Social Identity Model of Collective Action

According to the Social Identity Model of Collective Action (SIMCA), the identification with the protest group increases participation in collective actions. That is, people who identify with a protest group are motivated to engage in those protest actions enacted for or by that group (van Zomeren et al., 2012). Moreover, group identification indirectly predicts support for protest movements via feelings of group-based anger and a sense of group efficacy (van Zomeren et al., 2008). Group-based anger refers to those explanations based on perceived injustice, by which the perception of relative deprivation propels collective action (see Walker & Smith, 2002). Group efficacy refers to an instrumental explanation for collective action focusing on peoples’ expectations of whether the group action would be effective towards achieving its goal (see Mummendey, Kessler, Klink, & Mielke, 1999).

In order to investigate what motivates people to challenge social inequality regardless of whether they belong to the advantaged or disadvantaged group, van Zomeren et al. (2011, 2012) have recently taken into consideration the notion of moral convictions. As suggested by these authors, political action can be driven by group-based and personal moral motivations that reflect the goal of protecting or promoting some specific core values (van Zomeren, 2016). That is, political action is linked to subjectively absolutist stances on an issue and to a personal threshold for their violation, so-called moral convictions. By increasing identification with the protest group, moral convictions constitute a powerful engine for motivating collective action. In this sense, group identification provides “a conceptual bridge between seemingly individualistic moral convictions and group-based predictors of collective action identified by the Social Identity Model of Collective Action” (van Zomeren et al., 2011, p. 736).

However, moral convictions are measured as a violation of a specific moral norm connected to the protest in question. Therefore, they do not clarify why people hold such moral convictions in the first place. Where do specific moral convictions come from and what is the worldview behind such a moral activation? Could we connect them with specific ways of considering the relationship between the individual and the political system? By considering three distinct ways by which individuals relate to the political system, the orientations theorized by Kelman and Hamilton (1989) should explain the reasons why people consider that their moral conviction has been violated and consequently decide to join a protest action.

## The Three Political Orientations

The analysis of the distinct patterns of relationship with the political system by Kelman and Hamilton (1989) deals with the reasons behind the attachment to the political system and explains the motivations behind supporting or opposing the *status quo*. By examining the sources of the legitimacy of the system and its institutions, Kelman and Hamilton (1989) identify some individual differences in the way in which the political system is assessed and experienced. In particular, they recognised three types of political orientation of citizens to the political system: namely, rule-, role- and value-orientation. People who mainly focus on the social effects of their behavior exhibit some concern with how adequately they conform to societal rules (i.e., rule-oriented). People who mainly focus on the social anchorage and on social roles pay attention to how securely they are embedded in their positions within the society (i.e., role-oriented). Finally, people who mainly focus on a congruence with ethical values and principles place an emphasis on how fully they live up to societal values they share (i.e., value-oriented).

The three orientations characterise three ways by means of which the legitimacy of political authorities is generated, assessed and maintained by individuals. Although these orientations are not mutually exclusive—since they are likely to appear at some level in all individuals and they may coexist (Kelman & Hamilton, 1989)—they underline systematic differences in the way in which individuals define themselves as citizens and relate to the political system. That is, they support distinct beliefs as concerns the conception of authority and citizens’ responsibility, participation in performing citizenship duties, and the motivations to support or challenge authority’s policies and decisions. Specifically, for rule-oriented citizens their duties hinge on abiding by the rules and respecting authorities’ demands. In return, they expect authorities to enforce the rules, protect their basic interests and ensure societal order. Their participation tends to be passive and aims to protect their interests through a cost-benefit analysis (e.g., avoid penalties for rule-breaking). For role-oriented citizens, good citizens should meet their role obligation by actively and trustworthily obeying a government’s demands. In return, they expect authorities to uphold the integrity of their roles and to ensure their status. Their participation tends to be supportive, that is they are concerned with meeting their obligations as citizens by giving substantive support and taking an active part in implementing national policies (e.g., pursuing the affairs of a society). Finally, value-oriented citizens tend to be active in formulating, evaluating and questioning national policies. They expect both citizens and authorities to follow and enforce those policies that reflect the fundamental and universal values of a just and fair society^i^ (Fattori et al., 2015; Morselli & Passini, 2012, 2015; Passini & Morselli, 2010; Pozzi, Quartiroli, Alfieri, Fattori, & Pistoni, 2018).

These different ways of relating to political authorities translate into differences in the readiness to join collective actions for supporting or challenging an authority’s policies and decisions. Specifically, each orientation might lead to supporting protest actions when there is the perception that the contract with the political system has been or is likely to be violated. Given that rule-oriented citizens are sensitive to threats to their personal security, they will protest in the event of policies concerning personal security: i.e., contesting policies that they perceive as lessening their security or supporting collective actions to defend/enforce it. Some examples may be those protests against mass immigration or in favor of tougher sentencing for offenders. Role-oriented citizens will instead protest in case of policies concerning social status: that is, contesting policies that constitute a threat to their social position or supporting collective actions to defend/enforce it. Some examples may be those protests in defence of the privileges and benefits of a social group and against the enforcement of the same rights to other groups (e.g., protests that oppose the enactment of laws legalizing same-sex marriages). Finally, value-oriented citizens will be active in supporting protests concerning values of equality and fairness: that is, contesting policies that threaten values of social equality or supporting collective actions to defend/enforce them. Some examples may be those protests in favor of the extension of legal and civil rights to those social groups that were previously excluded (e.g., protests to grant citizenship rights to second-generation immigrants). Thus, each orientation should predict a willingness to attach importance to certain distinct moral convictions and therefore to be involved in distinct collective actions in their defense: that is, rule-oriented citizens should focus on convictions and engage in actions more related to personal security, role-oriented citizens on those concerning social roles, and finally value-oriented citizens on those more connected to social equality.

## Hypotheses

The aim of the present research is to analyze whether participation in collective action is influenced by the three individual orientations to the political system. In particular, the political orientations should explain why individuals hold a given attitude (positive or negative) towards a policy position, hold it with moral conviction, and decide to join a protest action. As outlined in the introduction, each political orientation is not affected by every protest issue and so they should predict collective action in distinct protest situations. Rule-oriented citizens should be involved in the event of policies concerning personal security and public order (e.g., a protest against an open-doors immigration policy); role-oriented citizens should be active in the case of policies concerning social status (e.g., a protest against the restriction of motor vehicle use during the week); and finally value-oriented citizens should be active in case of policies concerning values of equality and fairness (e.g., a protest against the restriction of press freedom).

In the present research, three protest scenarios were used. Study 1 comprised a Greenpeace protest against the non-visible use of cloned meat in consumer products. Study 2 comprised a scenario with two protest groups (a and b): that is, a law-enforcing (pro-status quo) *vs*. a human rights-protecting (pro-social change) collective action. Study 3 comprised another scenario with two protest groups (a and b): that is, a tradition-protecting (pro-status quo) *vs.* a human rights-protecting (pro-social change) collective action. Specifically, it is hypothesized that rule orientation should have a positive effect on collective action in those protests related to the protection of personal security and public order (i.e., Study 2a); role orientation should have a positive effect on collective action in those protests related to the safeguarding of one’s own social status (i.e., Study 3a); value-orientation should have a positive effect on collective action in those protests related to the safeguarding of social equality (i.e., Study 1, Study 2b and 3b). In Study 1, these effects are controlled for the effects of other variables linked to the individual-political system relationship, such as system justification, authoritarianism and social dominance orientation (SDO). In order to reduce the length of the questionnaire in Study 2 and 3, this hypothesis was tested only in Study 1.

## Analytical Procedures

Hypotheses were tested using path models analyzing the effect of the political orientations on the variables of SIMCA related to collective action (i.e., moral convictions, politicized identification, anger, efficacy, and protest intentions) via maximum likelihood estimation. Moreover, in Study 1 the same model with the addition of the effects of system justification, authoritarianism and SDO was tested. Consistent with the recommendation of Hu and Bentler (1999), goodness-of-fit criteria were used in order to quantify (relatively) good model fit (*CFI* > 0.95, *TLI* > 0.95, *SRMR* < 0.08, *RMSEA* < 0.06). Data were analyzed with Mplus 7.1 (Muthen & Muthen, 2012). Finally, the significance of indirect effects from the three political orientations on protest intentions through the mediating effects of moral convictions, politicized identification, anger, and efficacy was computed with Mplus (Delta Method; Taylor, MacKinnon, & Tein, 2008).

## Study 1

### Method

#### Participants

An online questionnaire was constructed using Limesurvey, a survey-generating tool (http://www.limesurvey.org). The questionnaire was publicly accessible and an invitation with the link to the questionnaire was emailed to the potential participants by various methods (e.g., mailing lists, newsgroups, social networking services). They were informed that their participation was voluntary and that their responses would be anonymous and confidential. No financial incentive was offered. The questionnaire was written in Italian. In order to check and prevent a person reentering the survey site, the subject’s IP address was monitored. The data were collected in 2015.

A total of 120 Italian citizens (64.2% women) responded by accessing the website and filling out the questionnaire. Participant ages ranged from 18 to 73 years (*M* = 41.56, *SD* = 13.71). As regards their level of education, 3.4% declared they had finished middle school, 39.3% declared they had earned a high school diploma, 47% had a university degree and 10.2% a master’s or Ph.D. qualification. Job-wise, 34% stated they were white collar workers, 16.5% university students, 4.9% factory workers/craftsmen, 9.7% self-employed, 11.7% teachers, 6.8% unemployed, 10.7% retired, and, finally, 5.7% chose “other.”

#### Procedure

The first section of the questionnaire contained the political orientation, system justification, authoritarianism and SDO scales. To avoid set responses, scale items were randomized each time for each participant by the computer program. Next, as in the study of van Zomeren et al. (2012), participants were introduced in the context of a Greenpeace protest against the non-visible use of cloned (i.e., genetically modified) meat in consumer products. They read a document stating that Italian universities and Greenpeace had joined forces in order to investigate what people think about the issue of cloned food. Some information about cloned food and about the plan of the European Committee to allow food producers to use cloned meat in their products without stating this clearly on food labels was provided. The protest thus concerned consumers’ “right to know” and should be supported by a value orientation because of its focus on the protection of basic individual rights. Afterwards, participants responded to the measures related to consumers’ “right to know” (i.e., variables measuring SIMCA).

#### Measures

All measures employed seven-point response scales (ranging from 1 = not at all to 7 = very much). Where not specified, the original English versions were translated into Italian and submitted to a back-translation by a native English speaker. The back-translated items were then reviewed by the authors and, where necessary, any unclear statement was reformulated. Scale reliabilities are shown in Appendix A.

##### Political orientation scale

A reduced form composed of 12 items of the political orientation scale constructed by Kelman and Hamilton (1989) was used. The original English 25-item version was reduced to a 12-item version by choosing for each of the three factors (i.e., rule, role, and value-orientation) the items that most adequately identify the orientation and with a high loading on the factor analysis computed by Kelman and Hamilton (1989, p. 284–285). The scale measures each of the three orientations (rule, role and value) with four items. This structure was upheld by a confirmatory factor analysis (CFA) computed with Mplus 7.1 (Muthen & Muthen, 2012). No cross-loadings were allowed. One item (i.e., “one should obey one's government, even when it means going against one's principles”) was dropped because it was not strongly associated with its factor (i.e., role orientation). The final three-factor model fit the data well (see Table 1): χ^2^(39) = 73.44, *CFI* = 0.94, *TLI* = 0.91, *RMSEA* = 0.06, *SRMR* = 0.06.

**Table 1:** Confirmatory Factor Analysis on the Political Orientation Scale on all the Studies

Item	Study		
	1	2	3
Rule orientation			
The government must sometimes set constitutional principles aside in order to deal with problems of the modern world.	.53	.85	.53
One reason for supporting the government is that anarchy will result if there are too many critics.	.56	.33	.54
The most you can expect the government to do is to see it that all citizens are able to earn a living and be safe.	.53	.68	.50
In matters involving the national interest, we cannot apply the same moral principles that we follow in everyday life.	.45	.27	.68
Role orientation			
A good Italian is one who is prepared to play his/her part in carrying out government policies.	.87	.62	.85
The most valuable contribution an individual citizen can make is to give active support to government policies.	.81	.50	.72
I feel an obligation to support the government even when its policies run counter to my values.	.55	.77	.67
Value orientation			
I feel obligated to protest both vigorously and publicly if the government does something that is morally wrong.	.69	.51	.62
I expect a good citizen to be politically aware and active all year long, not just around election time.	.62	.63	.76
The most valuable contribution an individual citizen can make is to maintain an active and questioning approach toward government policies.	.62	.55	.58
The most important thing the government can do is to see that the basic rights and freedoms of all citizens are protected.	.38	.47	.44

*Note.* All the items loaded on the hypothesized factor and were significant at *p <* .001. Loadings are standardized.

##### System justification

Participants completed the 8-item measure of the Italian version of the system justification scale, developed by Jost, Kivetz, Rubini, Guermandi, and Mosso (2005) which assesses “perceptions of the fairness, legitimacy, and justifiability of the prevailing social system” (Kay & Jost, 2003, p. 828). A sample item is “Society is set up so that people usually get what they deserve.”

##### Authoritarian submission

In the present research, only the authoritarian submission dimension was considered given that it is the dimension more pertinent to the attachment to the system and the relationship with the authority. This construct was measured by an Italian 4-item scale based on the Right-wing authoritarianism (RWA) by Altemeyer (1996), constructed and validated by Passini (2017). An example of an item is “Our country will be great if we do what the authorities tell us to do.”

##### Social Dominance Orientation (SDO)

Social dominance orientation was measured with the Italian 4-item version of the SDO scale (Pratto et al., 2013). A sample item of the scale is “Some groups of people are simply inferior to other groups.”

##### Moral convictions

Moral convictions were measured with three items: “My opinion on the right to know is an important part of my moral norms and values,” “My opinion on the right to know is a universal moral value that should apply everywhere in the world” and “My opinion on the right to know is a universal moral value that should apply at all times.”

##### Group-based anger

Group-based anger was measured with three items: “As a consumer, I feel angry/furious/outraged because of these European Committee plans.”

##### Group efficacy

Group efficacy was measured with three items: “As consumers, I think we can change these European Committee plans,” “As consumers, I think that we can successfully stop these European Committee plans” and “I think that, as consumers, we can change this situation together.”

##### Politicized identity

Politicized identity was measured with three items: “I see myself as a member of Greenpeace,” “I identify with Greenpeace members” and “I feel strong ties with Greenpeace.”

##### Protest intentions

Protest intentions were measured with four items: “I would participate in a demonstration against these European Committee plans,” “I would like to sign a petition against these European Committee plans,” “I would like to do something together against these European Committee plans” and “I would like to send a protest letter or an e-mail to the European Committee.”

### Results

**Figure 1:**
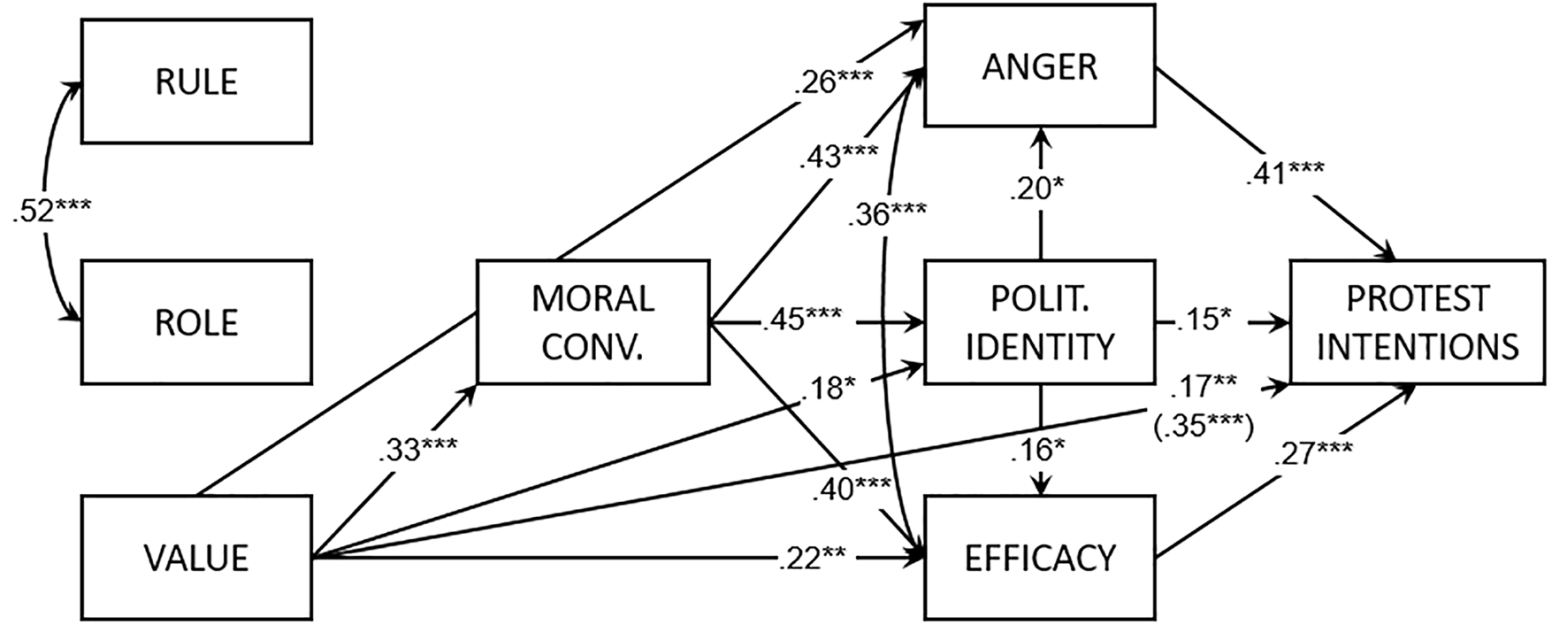
Path model of Study 1. *Note*. Reported coefficients are standardized. In parentheses, the indirect effect from the orientation to protest intentions. Polit. = Politicized.

Two participants were excluded because of missing values. The path model (see Figure 1) explained 66% of the variance of protest intentions: χ^2^(13) = 19.05, *CFI* = 0.98, *TLI* = 0.96, *RMSEA* = 0.06, *SRMR* = 0.07. As hypothesized, value orientation had a positive effect on moral conviction, anger, efficacy, identity, and protest intentions. Both rule and role orientation did not predict any variable. The mediation analysis showed a positive indirect effect of value orientation on protest intentions (*b* = .35, *SE* = 0.05, *t* = 5.93, *p* < .001). Rule and role orientations did not have a significant indirect effect on protest intentions.

System justification, authoritarianism, and SDO were inserted in the model, by setting free all the paths to the other variables. Non-statistically significant paths were therefore trimmed from the model. In the final model [χ^2^(34) = 52.02, *CFI* = 0.95, *TLI* = 0.92, *RMSEA* = 0.07, *SRMR* = 0.09], authoritarianism and SDO did not predict any variable and SDO had just a negative effect on anger (β = −.18, *p* < .01). Chi-square difference test indicated a non-significantly better fit over the previous model: Δχ^2^(−21) = −32.97, *p* = ns.

### Discussion

As hypothesized, in the case of a protest related to the protection of basic individual rights, value orientation has a both direct and indirect effect on collective action, while rule and role orientations do not. This is in line with the theory of Kelman and Hamilton (1989) who define this orientation to the political system as supporting protests in defending values of equality and fairness. Instead, rule and role orientations do not have an impact on this kind of protest, because the latter’s focus is separate from the reasons behind the adhesion to the political system.

Secondly, the other variables related to the relationship with the political system (i.e., system justification, authoritarianism, and SDO) are not predictive of collective action as value orientation is. Although these variables have a validity in explaining the individual’s attachment to the system and the support of hierarchy intergroup relations, in the case of the current protest scenario they do not sufficiently explain why people should engage, or not engage, in a protest action.

## Study 2

### Methods

#### Participants

The participants were contacted online using the same procedure as the previous study. A convenience sample of 213 Italian citizens (62.4% women) responded by accessing the website and filling out the questionnaire. The data were collected in 2014. Participant ages ranged from 19 to 67 years (*M* = 28.59, *SD* = 11.10). As regards their level of education, 3.3% declared they had finished middle school, 58.1% declared they had obtained a high school diploma, 38.1% had a university degree and 0.5% a master or a Ph.D. qualification. Job-wise, 56.1% stated they were university students, 13.8% white collar workers, 9.2% self-employed, 8.7% factory workers/craftsmen, 5.1% teachers, 4.1% unemployed, 2% retired, and, finally, 1% chose “other.”

#### Procedure

Participants firstly responded to the political orientation scale. Next, the protest scenario was introduced by a make-believe document affirming that the Italian parliament is debating whether or not to pass a law on the right to host migrants to regulate the migration flow, to restrain the Italian Navy’s actions to send back them and to improve the living conditions of immigrants held in detention centers. Then, on the basis of their month of birth, participants were randomly assigned to two different protest groups. The first one (*n* = 97) was a law-enforcing protest group: “the Citizens Commission for Security is organizing a demonstration in defense of the current laws and in defense of personal and job security of Italian citizens. The Committee demands more effective supervision of our borders by strengthening the Navy’s actions.” This protest group should be supported by a rule orientation because of its focus on policies concerning personal security. The second one (*n* = 116) was a human rights-protecting protest group: “the Committee of Citizens for Human Rights is organizing a demonstration to promote the right to host migrants in order to urge parliament to approve this law in a short time.” This protest group should be supported by a value orientation because of its focus on policies concerning values of social equality.

All measures employed seven-point response scales (ranging from 1 = not at all to 7 = very much). Scale reliabilities are shown in Appendix B. All the measures used were the same of Study 1, adapted for the context of the protest scenario. Examples of items are: “My opinion on the [security of Italian citizens *vs.* the right to host migrants] is an important part of my moral norms and values” (moral convictions); “I feel angry [for the proposal to pass *vs*. because we do not proceed to the approval of] a law to host migrants” (group-based anger); “I think that together we can successfully fight [against *vs.* for] the approval of a law to host migrants” (group efficacy); “I see myself as a member of the [Citizens Commission for Security *vs*. Committee of Citizens for Human Rights]” (politicized identity); “I would participate in a demonstration [against *vs.* in favor of] the approval of a law to host migrants” (protest intentions). As concerns the political orientation scale, the three-factor model fit the data well (see Table 1): χ^2^(39) = 73.44, CFI = 0.93, TLI = 0.90, RMSEA = 0.06, SRMR = 0.06.

### Results

**Figure 2:**
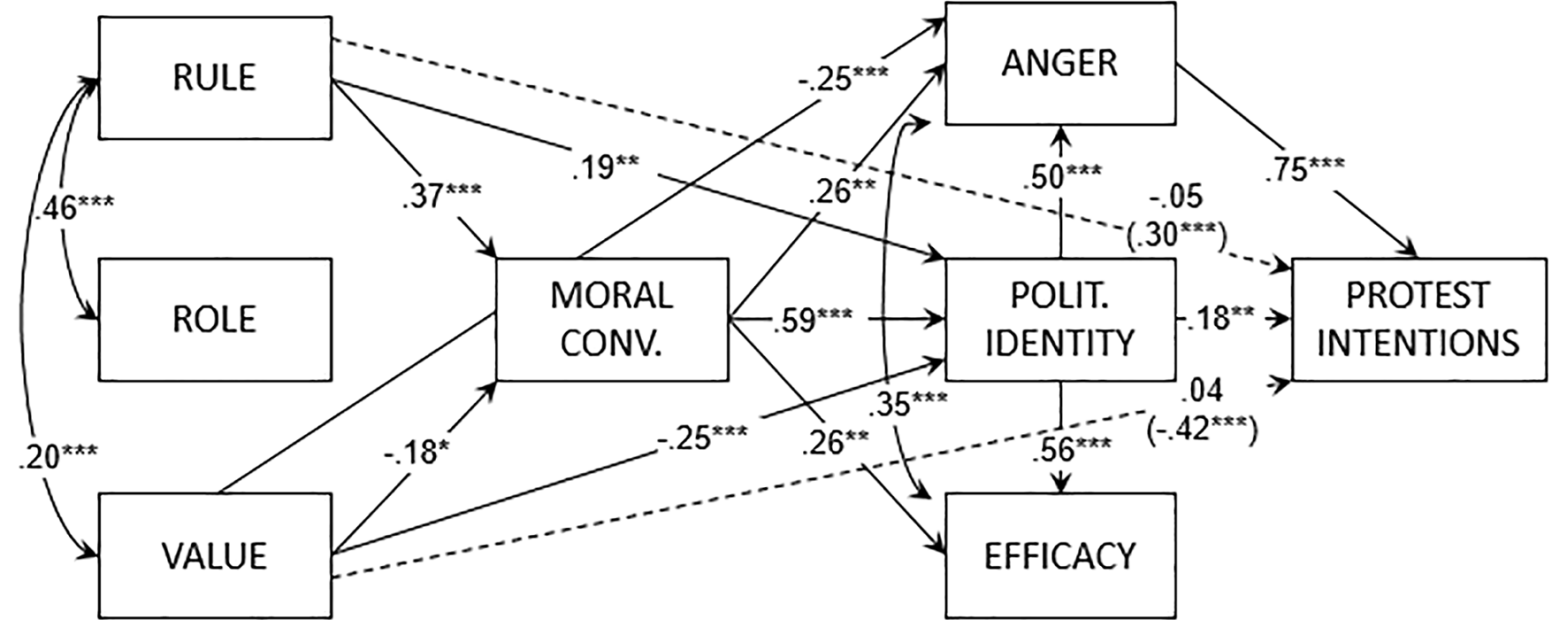
Path model of Study 2 (law-enforcing protest group). *Note*. Reported coefficients are standardized. In parentheses, the indirect effects from the orientations to protest intentions. Polit. = Politicized.

None of the participants had missing values. The path models explained 80% of the variance in protest intentions for the law-enforcing protest group [χ^2^(13) = 16.35, *CFI* = 0.99, *TLI* = 0.99, *RMSEA* = 0.03, *SRMR* = 0.05] and 89% for the human rights-protecting protest group [χ^2^(16) = 21.60, *CFI* = 0.99, *TLI* = 0.99, *RMSEA* = 0.04, *SRMR* = 0.04]. For the law-enforcing protest group (see Figure 2), rule orientation had a positive effect on moral conviction and politicized identity, while value orientation had a negative effect on moral conviction, politicized identity, and anger. Role orientation did not predict any variable. The mediation analysis showed a positive indirect effect of rule orientation (*b* = .30, *SE* = 0.06, *t* = 4.80, *p* < .001) and a negative indirect effect of value orientation (*b* = −.42, *SE* = 0.07, *t* = −6.18, *p* < .001) on protest intentions. Role orientation did not have a significant indirect effect on protest intentions.

**Figure 3:**
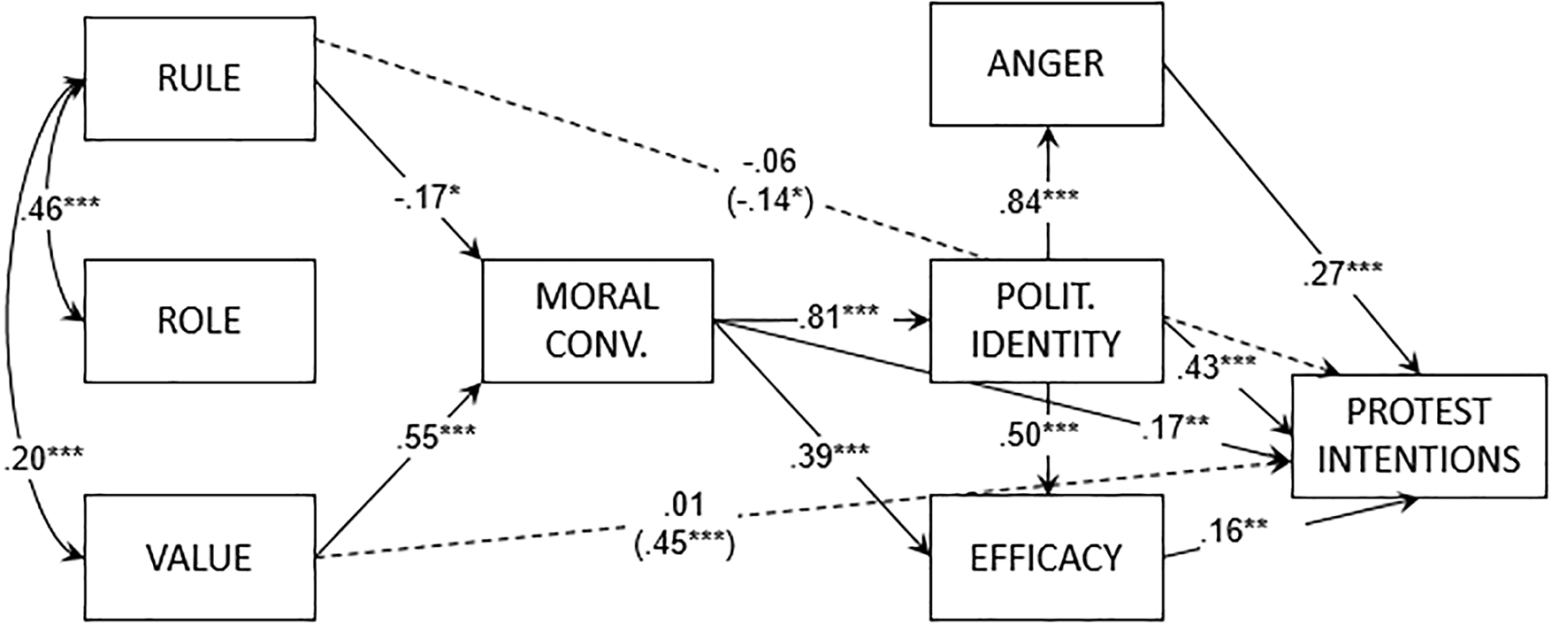
Path model of Study 2 (human rights-protecting protest group). *Note*. Reported coefficients are standardized. In parentheses, the indirect effects from the orientations to protest intentions. Polit. = Politicized.

For the human rights-protecting protest group, the path model (see Figure 3) showed a positive effect of value orientation on moral conviction and a negative effect of rule orientation on it. Role orientation did not predict any variable. The mediation analysis showed a positive indirect effect of value orientation (*b* = .45, *SE* = 0.06, *t* = 7.39, *p* < .001) and a negative indirect effect of rule orientation (*b* = −.14, *SE* = 0.07, *t* = −2.13, *p* < .05) on protest intentions. Role orientation did not have a significant indirect effect on protest intentions.

### Discussion

As hypothesized, rule and value orientations have a direct effect on moral convictions and an indirect effect on protest intentions depending on the specific protest condition: that is, rule orientation has positive effects in those protests reinforcing security and public order, while value orientation has positive effects in those protests in favor of the enlargement of social equality. Moreover, the two orientations have also negative effects on moral convictions and protest intentions for the other protest group: specifically, rule orientation against open-door immigration policies and value orientation against curbing immigrants’ rights.

## Study 3

### Methods

#### Participants

The participants were contacted online using the same procedure as the previous study. A convenience sample of 266 Italian citizens (53% women) responded by accessing the website and filling out the questionnaire. The data were collected in 2015. Participant ages ranged from 18 to 70 years (*M* = 35.51, *SD* = 13.79). As regards their level of education, 11.6% declared they had finished middle school, 53.8% declared they had obtained a high school diploma, 30.4% had a university degree and 4.2% a master or a Ph.D. qualification. Job-wise, 30% stated they were university students, 23.8% white collar workers, 11.9% teachers, 11.5% factory workers/craftsmen, 8.4% self-employed, 5.3% retired, 4.8% unemployed, and, finally, 4.3% chose “other.”

#### Procedure

Participants first of all responded to the political orientation scale. Afterwards, they read a make-believe document stating that the Italian parliament is discussing as to whether or not to pass a law on civil unions, that is, on the civil and legal recognition of unmarried couples, whether heterosexual or homosexual, and its equivalence to couples united through the marriage. Then, depending on their month of birth, participants were randomly assigned to two different protest groups. The first one (*n* = 111) was a tradition-protecting protest group: “the Forum of the Family Associations is organizing a demonstration in defense of the traditional family model, established and legitimized by the marriage between a man and a woman, as opposed to the legal recognition of unmarried couples.” This protest group should be supported by a role orientation because of its focus on policies concerning the defence of one’s own social status. The second one (*n* = 155) was instead a human rights-protecting protest group: “the group ‘Civil Unions for Everyone’ is organizing an event to promote the rights of unmarried couples, both heterosexual and homosexual, as opposed to the maintenance of the legal recognition to traditional marriages alone.” This protest group should be supported by a value orientation because of its focus on policies concerning values of social equality.

All measures employed seven-point response scales (ranging from 1 = not at all to 7 = very much). Scale reliabilities are shown in Appendix C. All the measures used were the same of Study 1 and 2, adapted for the context of the protest scenario. Examples of items are: “My opinion on the [defense of the traditional family model *vs.* the rights of unmarried couples] is an important part of my moral norms and values” (moral convictions); “I feel angry [for the proposal of *vs*. because we do not proceed with] the legal recognition of unmarried couples” (group-based anger); “I think that together we can successfully fight [against *vs.* for] the legal recognition of unmarried couples” (group efficacy); “I see myself as a member of the [Forum of the Family Associations *vs*. Civil Unions for Everyone]” (politicized identity); “I would participate in a demonstration [against *vs.* in favor of] the legal recognition of unmarried couples” (protest intentions). As concerns the political orientation scale, the three-factor model fit the data well (see Table 1): χ^2^(36) = 67.35, *CFI* = 0.97, *TLI* = 0.96, *RMSEA* = 0.05, *SRMR* = 0.05.

### Results

**Figure 4:**
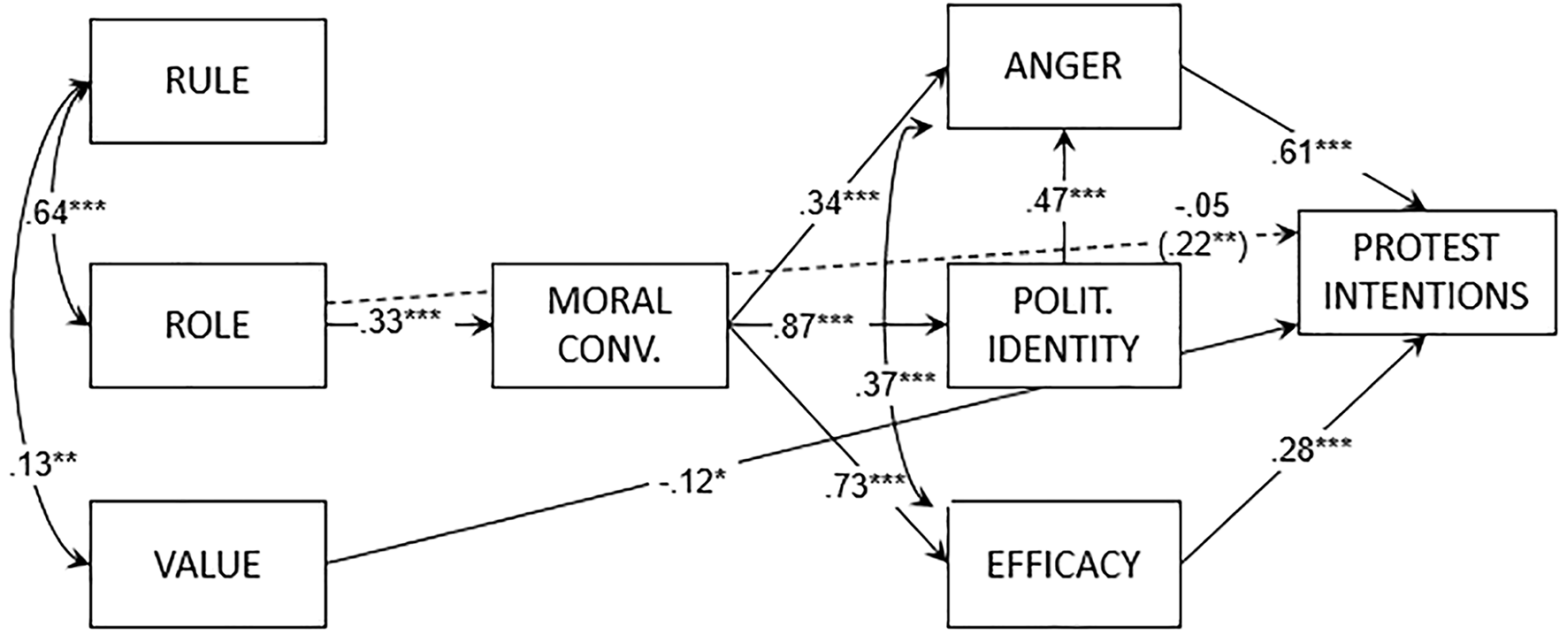
Path model of Study 3 (tradition-enhancing protest group). *Note*. Reported coefficients are standardized. In parentheses, the indirect effect from the orientation to protest intentions. Polit. = Politicized.

None of the participants had missing values. The path models explained 71% of the variance in protest intentions for the tradition-enforcing protest group [χ^2^(17) = 23.52, *CFI* = 0.99, *TLI* = 0.98, *RMSEA* = 0.04, *SRMR* = 0.06] and 87% for the human rights-protecting protest group [χ^2^(18) = 34.68, *CFI* = 0.98, *TLI* = 0.97, *RMSEA* = 0.06, *SRMR* = 0.05]. As hypothesized, for the tradition-enhancing protest group (see Figure 4) role orientation had a positive effect on moral conviction. Moreover, value orientation had a negative effect on protest intentions. Rule orientations did not predict any variable. The mediation analysis showed a positive indirect effect of role orientation (*b* = .22, *SE* = 0.06, *t* = 3.80, *p* < .001) on protest intentions. Rule and value orientations did not have a significant indirect effect on protest intentions.

**Figure 5:**
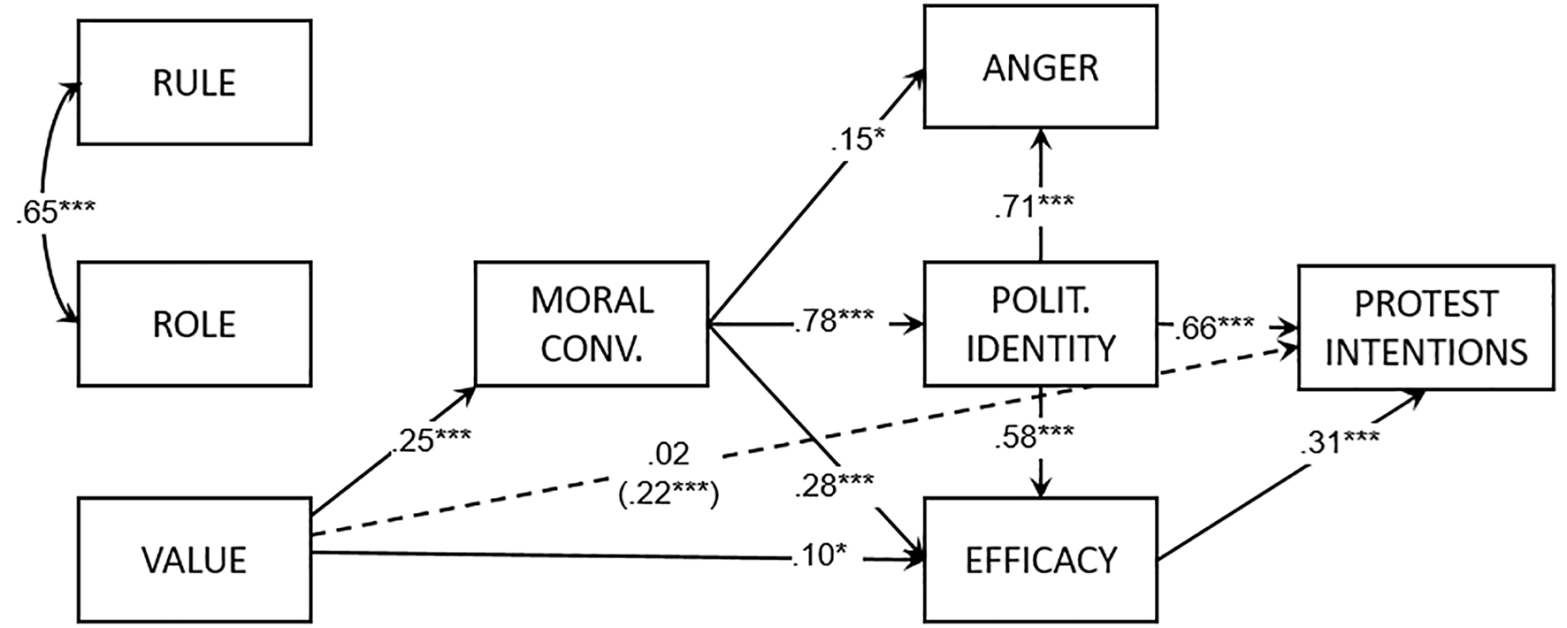
Path model of Study 3 (human rights-protecting protest group). *Note*. Reported coefficients are standardized. Between parentheses, the indirect effect from the orientation to protest intentions. Polit. = Politicized.

For the human rights-protecting protest group (see Figure 5), the path model showed a positive effect of value orientation on moral conviction and group efficacy. Rule and role orientations did not predict any variable. The mediation analysis showed a positive indirect effect of value orientation (*b* = .22, *SE* = 0.06, *t* = 3.69, *p* < .001) on protest intentions. Rule and role orientations did not have a significant indirect effect on protest intentions.

### Discussion

As hypothesized, role and value orientations have a direct effect on moral convictions and an indirect effect on protest intentions depending on the protest group analyzed. Role orientation has positive effects on those protest related to the defense of the *status quo* and of the traditional family model. This protest was conceived by participants as a support for the protection of one’s own social status and privileges, like those derived from the established civil rights for married couples. Protest in favor of the rights of unmarried couples (both heterosexual and homosexual) were instead predicted by the value orientation, as a confirmation of a focus of this orientation on the broadening of civil rights and social equality. Moreover, value orientation also negatively predicts the involvement in protests against these rights.

## General Discussion

The aim of the present research was to extend the analysis of the motivations that drive people to participate in social protests both against and in favor of the *status quo*, by considering the three political orientations detected by Kelman and Hamilton (1989). By exploring the different ways people have of feeling loyalty to the political system, this theory should explain the reasons why people hold a positive or negative attitude towards a policy position, hold it with moral conviction, and decide to join a protest action.

First, results of all the three studies show a distinct effect of each political orientation on moral convictions (direct effect) and intention to protests (indirect effect), depending on the aim of the protest. That is, protests reinforcing personal security and law and order are mainly pursued by rule-oriented citizens; those linked to one’s social status by role-oriented citizens; and finally those focused on social equality by value-oriented citizens. In particular, the path models show a direct connection between the three orientations and the specific moral convictions related to the protest action in question. The three orientations explain why these convictions are central to one’s own sense of justice to the point that a person decides to protest in favor or against the status quo. Moreover, each orientation indirectly predicts the intention to engage in the protest. These indirect effects are mediated by the other variables of SIMCA, that is, moral convictions, politicized identification, anger, and efficacy. It is worthwhile noting that while all these variables are, by definition, closely related to the single protest considered, the three orientations measure a more general opinion in respect to the relationship between the individual and the political system. In this way, the three orientations identified by Kelman and Hamilton (1989) further clarify the understanding of the reasons why certain moral convictions are considered as violated, why people identify with a protest group and why they decide to participate in their collective action as a consequence.

Second, results of all the studies confirm the validity of the SIMCA in explaining protest intentions in case of a social equality’s violation scenario. Indeed, moral convictions and identification with the protest group have an effect on protest intentions via group-based anger and group efficacy. In the case of a protest scenario in defense of the *status quo*, the SIMCA is not totally confirmed as politicized identity is not a robust predictor of protest intentions. This result suggests that, in cases of a protest in support of the *status quo*, motivation to protest is more related to a sense of anger and group efficacy than to the identification with the protest group (see Reichert, 2016). It can be supposed that individuals do not need to identify with a specific group as the position is the one held by the authority and the political system itself. Future studies should analyze this lack of effect of politicized identity in explaining pro-authority scenarios.

### Limitations and Directions for Future Research

This research has some limitations that can be overcome in future studies. First, future research should deepen the relationship between political orientations and the violation of moral convictions. For instance, it should be assessed whether an orientation facilitates how easily individuals perceive their moral convictions are being violated and in which cases. Experimental designs (e.g., explicit violation *vs.* no explicit violation) may deepen this issue. Second, it may be interesting to explore why the population at times pays attention to a minority’s claim and why at other times this fails to occur (Passini & Morselli, 2013, 2015). It may be interesting to consider the role of media and political parties in directing attention to one issue rather than another. Third, it may well be interesting to involve participants who are political activists (e.g., Morgan & Chan, 2016), in order to see whether their involvement may strengthen or weaken the models.

Despite these limitations, the results presented in this article provide some novel considerations into the social-psychological analysis of the motivations behind protest actions. Specifically, they improve the understanding of why some people engage in protest actions supporting the status quo while other people support collective actions against it. The focus on the social contract that defines the reciprocal relationships between individuals and the authority provided by the theory of Kelman and Hamilton (1989) improves the understanding of the reasons why people consider this contract to be undermined by some policies and why they are motivated to protest against them. To sum up, the present research suggests that people protest because of a view of the society in which certain violations run counter to the principles underpinning the contract with the political system.

## Appendix

**Appendix A:** Pearson Correlation Coefficients Among all the Variables (Study 1)

Measure	1	2	3	4	5	6	7	8	9	10	11
1. Orientation rule	.*63*										
2. Orientation role	.52***	.*77*									
3. Orientation value	.08	.11	.*66*								
4. System justification	.14	.29**	−.19*	.*75*							
5. Authoritarianism	.48***	.45***	−.14	.22*	.*68*						
6. SDO	.18*	.15	−.19*	.24**	.35***	.*71*					
7. Moral convictions	−.02	.12	.33***	−.02	−.05	−.15	.*88*				
8. Anger	−.10	.05	.47***	−.21*	−.18	−.34***	.62***	.*89*			
9. Efficacy	.06	.17	.40***	−.07	−.05	−.20*	.56***	.65***	.*89*		
10. Politicized identity	.19	.22*	.34***	−.01	.02	−.14	.51***	.52***	.44***	.*94*	
11. Protest intention	−.06	.10	.53***	−.16	−.18	−.28**	.53***	.74***	.67***	.54***	.*87*

*Note.* SDO = Social dominance orientation. All the variables extended from 1 to 7. Cronbach’s α in italics on the diagonal.

**p* < .05. ***p* < .01. ****p* < .001.

**Appendix B:** Pearson Correlation Coefficients Among all the Variables (Study 2)

Measures	1	2	3	4	5	6	7	8
1. Orientation rule	.*62*	.47***	.24***	−.02	−.01	.03	−.03	−.04
2. Orientation role	.47***	.*75*	.10	−.01	−.04	−.05	−.01	.03
3. Orientation value	.24***	.10	.*62*	.51***	.31***	.45***	.39***	.39***
4. Moral conviction	.34***	.28**	−.11	*.80/.87*	.73***	.79***	.80***	.83***
5. Anger	.30**	.28**	−.40***	.64***	*.89/.88*	.74***	.84***	.86***
6. Efficacy	.32***	.29**	−.08	.64***	.70***	*.81/.87*	.82***	.84***
7. Politicized identity	.35***	.24*	−.28**	.68***	.76***	.74***	*.89/.88*	.91***
8. Protest intention	.28**	.30**	−.31**	.63***	.89***	.70***	.75***	*.91/.90*

*Note.* All the variables extended from 1 to 7. Values below the diagonal are for the law-enforcing protest group. Values above the diagonal are for the human rights-protecting protest group. Cronbach’s α in italics on the diagonal.

**p* < .05. ***p* < .01. ****p* < .001.

**Appendix C:** Pearson Correlation Coefficients Among all the Variables (Study 3)

Measures	1	2	3	4	5	6	7	8
1. Orientation rule	.*69*	.65***	.20***	−.02	.10	−.07	−.04	−.08
2. Orientation role	.65***	.*78*	.12	−.08	.01	−.16*	−.07	−.15*
3. Orientation value	.21***	.12	.*69*	.25**	.17*	.30***	.23**	.25**
4. Moral conviction	.26**	.34***	−.05	*.94/.81*	.70***	.75***	.78***	.75***
5. Anger	.21*	.30***	−.16	.75***	*.92/.89*	.70***	.82***	.78***
6. Efficacy	.19*	.32***	−.11	.73***	.73***	*.76/.91*	.81***	.85***
7. Politicized identity	.28**	.37***	−.05	.87***	.79***	.70***	*.92/.92*	.92***
8. Protest intention	.19*	.25**	−.22*	.64***	.82***	.73***	.68***	*.81/.90*

*Note.* All the variables extended from 1 to 7. Values below the diagonal are for the tradition-protecting protest group. Values above the diagonal are for the human rights-protecting protest group. Cronbach’s α in italics on the diagonal.

**p* < .05. ***p* < .01. ****p* < .001.

## References

[r1] Altemeyer, B. (1996). *The authoritarian specter*. Cambridge, MA, USA: Harvard University Press.

[r2] Fattori F. Curly S. Jörchel A. C. Pozzi M. Mihalits D. Alfieri S. (2015). Authority relationship from a societal perspective: Social representations of obedience and disobedience in Austrian young adults. *Europe’s Journal of Psychology*, 11(2), 197–213. doi: 10.5964/ejop.v11i2.883 PMC4873106 27247652

[r3] Hu L. Bentler P. M. (1999). Cutoff criteria for fit indexes in covariance structure analysis: Conventional criteria versus new alternatives. *Structural Equation Modeling: A Multidisciplinary Journal*, 6(1), 1–55. doi: 10.1080/10705519909540118

[r4] Jost J. T. Kivetz Y. Rubini M. Guermandi G. Mosso C. (2005). System-justifying functions of complementary regional and ethnic stereotypes: Cross-national evidence. *Social Justice Research*, 18(3), 305–333.

[r5] Kay A. C. Jost J. T. (2003). Complementary justice: Effects of “poor but happy” and “poor but honest” stereotype exemplars on system justification and implicit activation of the justice motive. *Journal of Personality and Social Psychology*, 85(5), 823. doi: 10.1037/0022-3514.85.5.823 14599247

[r6] Kelman, H. C., & Hamilton, V. L. (1989). *Crimes of obedience. Toward a social psychology of authority and responsibility*. New Haven, CT, USA: Yale University Press.

[r7] Morgan G. S. Chan W. Y. (2016). Different paths to protest: Predictors of collective action in the Occupy Movement. *Journal of Applied Social Psychology*, 46(10), 565–582. doi: 10.1111/jasp.12386

[r8] Morselli D. Passini S. (2010). Avoiding crimes of obedience: A comparative study of the autobiographies of Gandhi, Nelson Mandela, and Martin Luther King, Jr. *Peace and Conflict: Journal of Peace Psychology*, 16(3), 295–319. doi: 10.1080/10781911003773530

[r9] Morselli D. Passini S. (2011). New perspectives on the study of the authority relationship: Integrating individual and societal level research. *Journal for the Theory of Social Behaviour*, 41(3), 291–307.

[r10] Morselli D. Passini S. (2012). Disobedience and support for democracy: Evidences from the World Values Survey. *The Social Science Journal*, 49(3), 284–294. doi: 10.1016/j.soscij.2012.03.005

[r11] Morselli D. Passini S. (2015). Value-oriented citizenship index: New extensions of Kelman and Hamilton’s theory to prevent autocracy. *Social Science Research*, 54, 289–302. doi: 10.1016/j.ssresearch.2015.08.006 26463549

[r12] Mummendey A. Kessler T. Klink A. Mielke R. (1999). Strategies to cope with negative social identity: Predictions by social identity theory and relative deprivation theory. *Journal of personality and social psychology*, 76(2), 229. 10074707 10.1037//0022-3514.76.2.229

[r13] Muthen, L. K., & Muthen, B. (2012). *Mplus: Statistical analysis with latent variables*. Los Angeles, CA, USA: Muthén & Muthén.

[r14] Passini S. (2017). Different ways of being authoritarian: The distinct effects of authoritarian dimensions on values and prejudice. *Political Psychology*, 38(1), 73–86. doi: 10.1111/pops.12309

[r15] Passini S. Morselli D. (2010). Disobeying an illegitimate request in a democratic or authoritarian system. *Political Psychology*, 31(3), 341–355. doi: 10.1111/j.1467-9221.2010.00761.x

[r16] Passini S. Morselli D. (2013). The triadic legitimacy model: Understanding support to disobedient groups. *New Ideas in Psychology*, 31(2), 98–107. doi: 10.1016/j.newideapsych.2012.06.002

[r17] Passini S. Morselli D. (2015). Supporting protest movements: The effect of the legitimacy of the claims. *International Journal of Psychological Research*, 8(2), 10–22.

[r18] Pozzi M. Quartiroli A. Alfieri S. Fattori F. Pistoni C. (2018). (Dis)Obedience in U.S. American young adults: A new way to describe authority relationships. *Europe’s Journal of Psychology*, 14(2), 404–423. doi: 10.5964/ejop.v14i2.1314 PMC6016029 30008954

[r19] Pratto F. Çidam A. Stewart A. L. Zeineddine F. B. Aranda M. Aiello A. et al. (2013). Social dominance in context and in individuals contextual moderation of robust effects of social dominance orientation in 15 languages and 20 countries. *Social Psychological and Personality Science*, 4(5), 587–599. doi: 10.1177/1948550612473663

[r20] Reichert F. (2016). How internal political efficacy translates political knowledge into political participation. *Europe’s Journal of Psychology*, 12(2), 221–241. doi: 10.5964/ejop.v12i2.1095 PMC4894288 27298633

[r21] Simon B. Klandermans B. (2001). Politicized collective identity: A social psychological analysis. *American Psychologist*, 56(4), 319–331. doi: 10.1037/0003-066X.56.4.319 11330229

[r22] Taylor A. B. MacKinnon D. P. Tein J.-Y. (2008). Tests of the three-path mediated effect. *Organizational Research Methods*, 11(2), 241–269.

[r23] van Zomeren M. (2016). Building a tower of babel ? Integrating core motivations and features of social structure into the political psychology of political action. *Advances in Political Psychology*, 37, 87–114. doi: 10.1111/pops.12322

[r24] van Zomeren M. Postmes T. Spears R. (2008). Toward an integrative social identity model of collective action: A quantitative research synthesis of three socio-psychological perspectives. *Psychological Bulletin*, 134(4), 504–535. doi: 10.1037/0033-2909.134.4.504 18605818

[r25] van Zomeren M. Postmes T. Spears R. (2012). On conviction’s collective consequences: Integrating moral conviction with the social identity model of collective action. *British Journal of Social Psychology*, 51(1), 52–71. doi: 10.1111/j.2044-8309.2010.02000.x 22435846

[r26] van Zomeren M. Postmes T. Spears R. Bettache K. (2011). Can moral convictions motivate the advantaged to challenge social inequality? Extending the social identity model of collective action. *Group Processes & Intergroup Relations*, 14(5), 735–753. doi: 10.1177/1368430210395637

[r27] Walker, I., & Smith, H. J. (2002). *Relative deprivation: Specification, development, and integration*. Cambridge, GBR: Cambridge University Press.

[r28] Willis-Esqueda C. Delgado R. H. Pedroza K. (2017). Patriotism and the impact on perceived threat and immigration attitudes. *The Journal of Social Psychology*, 157(1), 114–125. doi: 10.1080/00224545.2016.1184125 27136269

[r29] Wisneski D. C. Skitka L. J. (2017). Moralization through moral shock: Exploring emotional antecedents to moral conviction. *Personality and Social Psychology Bulletin*, 43(2), 139–150. doi: 10.1177/0146167216676479 27872393

